# Survival prediction for patients with non-resectable intrahepatic cholangiocarcinoma undergoing chemotherapy: a retrospective analysis comparing the tumor marker CA 19-9 with cross-sectional imaging

**DOI:** 10.1007/s00432-020-03200-2

**Published:** 2020-03-30

**Authors:** Felix Hahn, Lukas Müller, Florian Jungmann, Aline Mähringer-Kunz, Yasemin Tanyildizi, Christoph Düber, Peter R. Galle, Arndt Weinmann, Roman Kloeckner

**Affiliations:** 1 Department of Diagnostic and Interventional Radiology, University Medical Center of the Johannes Gutenberg-University Mainz, Langenbeckst. 1, 55131 Mainz, Germany; 2 Department of Neuroradiology, University Medical Center of the Johannes Gutenberg-University Mainz, Mainz, Germany; 3 Department of Internal Medicine, University Medical Center of the Johannes Gutenberg-University Mainz, Mainz, Germany; 4 Clinical Registry Unit (CRU), University Medical Center of the Johannes Gutenberg-University Mainz, Mainz, Germany

**Keywords:** Intrahepatic cholangiocarcinoma, Chemotherapy, Survival prediction, RECIST 1.1

## Abstract

**Purpose:**

Carbohydrate antigen (CA) 19-9 has been established as the main serum marker for patients with intrahepatic cholangiocarcinoma (ICC). The aim of this study was to compare the prognostic value of CA 19-9 changes versus response determined by imaging in patients with ICC undergoing chemotherapy.

**Methods:**

Between 2003 and 2018, 151 patients with histopathologically confirmed ICC underwent chemotherapy at our tertiary care center for non-resectable or recurrent ICC, of whom 121 were included in this study. Serum CA 19-9 levels and imaging were retrospectively evaluated during chemotherapy. Log-rank testing and optimal stratification were used to classify patients into risk groups.

**Results:**

Prior to chemotherapy, baseline serum CA 19-9 levels above the previously published cut-off of 37 U/ml were associated with poor survival (median OS 8.7 vs. 12.4 months, *p* = 0.003). After the beginning of chemotherapy, an increase in CA 19-9 of more than 40 U/ml resulted in impaired residual survival (median OS 5.0 vs. 12.1 months, *p* < 0.001). However, progressive disease at the first follow-up imaging proved the strongest predictor for poor outcome (median OS 4.6 vs. 15.5 months, *p* < 0.001). In contrast to prior studies, our data did not show statistically relevant differences in survival time with respect to absolute or relative decreases in serum CA 19-9 levels.

**Conclusion:**

In our study, the disease control rate—that is, the absence of progressive disease—was the strongest predictor of prolonged residual OS. To this end, both CA 19-9 changes and progressive disease on initial follow-up showed remarkable discriminatory power, with the latter slightly outperforming the former. Therefore, imaging should remain the mainstay of patient evaluation during follow-up.

**Electronic supplementary material:**

The online version of this article (10.1007/s00432-020-03200-2) contains supplementary material, which is available to authorized users.

## Introduction

Intrahepatic cholangiocarcinoma (ICC) is the second-most common primary liver malignancy. Its incidence has increased markedly over the past three decades and is estimated to be around 0.4–2.0/100,000 in the low endemic western countries (Hahn et al. 2011; Petrick et al. 2016; Shaib et al. 2004; Yang et al. 2012).

Unfortunately for the affected patients, this tumor type is often at an advanced stage at the time of diagnosis. Resection is the only curative treatment option (Guro et al. 2017). Even if resection is possible, the recurrence rate is high and recurrence is reported in up to two-thirds of patients (Park et al. 2016). For patients for whom resection is either initially or in the course of the disease no longer possible, different chemotherapy regimens have been proposed. Since the publication of the multicenter UK-ABC 02 study in 2010, the combination of gemcitabine and cisplatin has been widely administered as first-line chemotherapy (Valle et al. 2010). Systemic chemotherapy is the mainstay of treatment, but in the last decade transarterial therapies such as transarterial chemo-embolization (TACE) and selective internal radiation therapy (SIRT) have also become treatment options for selected patients (Boehm et al. 2015). However, the prognosis for ICC patients remains poor (Weber et al. 2015).

Carbohydrate antigen (CA) 19-9 is the main serum tumor marker for patients with ICC (Brito et al. 2015). Besides having diagnostic value, CA 19-9 has repeatedly been shown to be useful in predicting the overall survival (OS) or progression-free survival of patients with ICC. However, there is disagreement about the optimal cut-off value that identifies patients with poor prognosis, with proposed cut-offs ranging from 37 to 1000 U/ml (Ali et al. 2007; Bolm et al. 2019; Coelho et al. 2017; He et al. 2018; Jiang et al. 2011). Regarding the influence of CA 19-9 changes on survival in patients undergoing chemotherapy, a literature search yielded only two studies that focused on this issue. The first, by Harder et al., postulates that a decrease in CA 19-9 under chemotherapy is prognostically favorable, without giving a specific cut-off value (Harder et al. 2007). In the second study, by Lee et al., a relative decrease in CA 19-9 of more than 50% after two cycles of gemcitabine-based chemotherapy was associated with prolonged survival (Lee et al. 2016).

Apart from tumor markers, cross-sectional imaging is the standard of care to monitor treatment response in cancer patients. The response evaluation criteria in solid tumors (RECIST) objectively classifies response into complete response (CR), partial response (PR), stable disease (SD), and progressive disease (PD) (Eisenhauer et al. 2009). Moreover, imaging also provides information about whether progress is due to local tumor advancement or metastatic spread, and treatment adjustments can be initiated depending on imaging results.

The aim of this study was to compare the discriminatory power of CA 19-9 changes versus response determined by imaging to identify patients at risk while receiving palliative chemotherapy.

## Material and methods

**Fig. 1:**
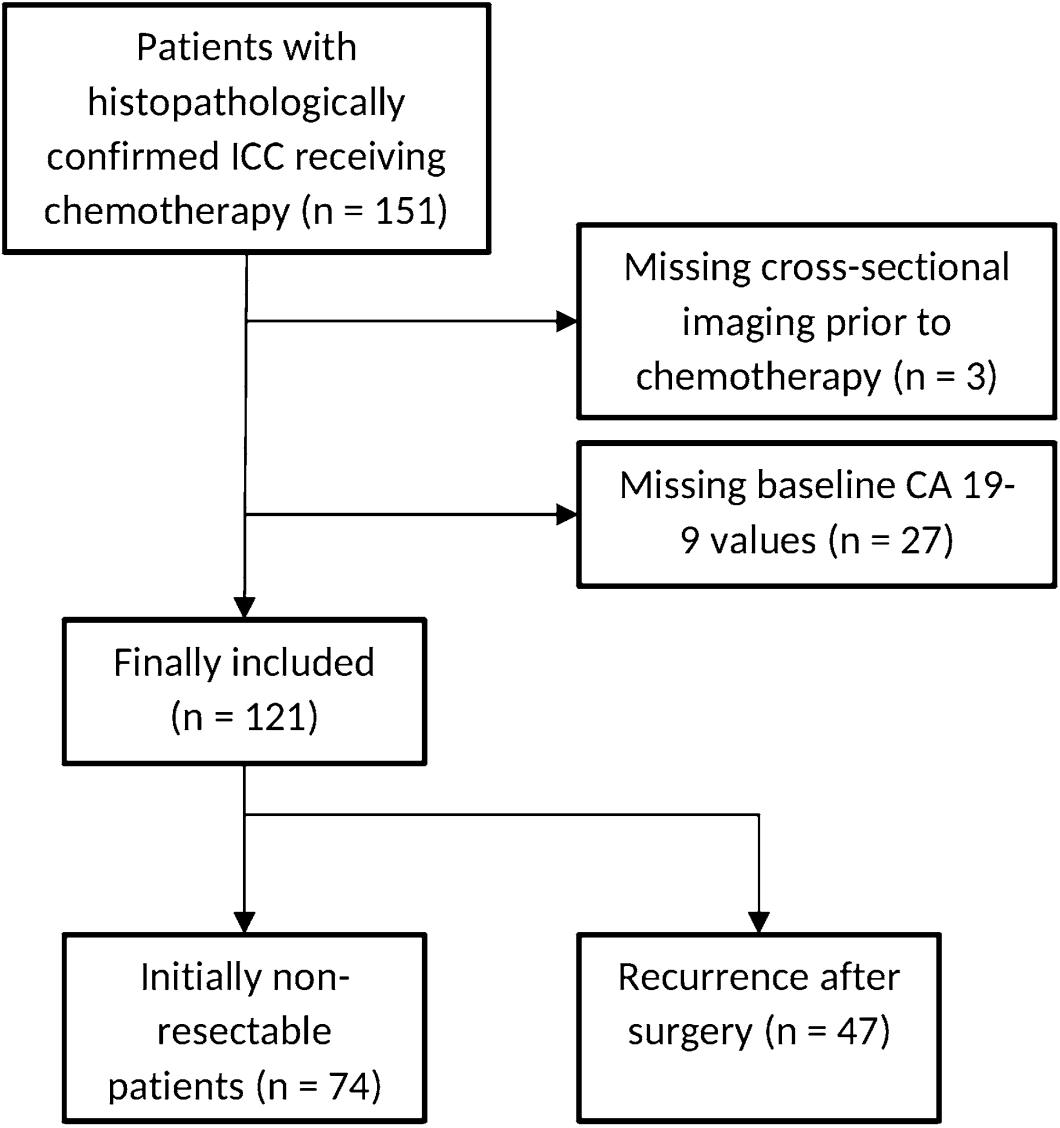
STROBE flow chart showing the number of patients included in this study (CA 19–9, carbohydrate antigen 19–9)

Between January 2003 and February 2018, 151 patients with histopathologically confirmed ICC were treated with chemotherapy for non-resectable or recurrent ICC at our tertiary care center. Follow-up ended on February 28, 2019. The patients were retrospectively identified out of a prospectively maintained clinical registry unit. Of the 151 patients, cross-sectional imaging prior to chemotherapy was missing in three patients. Baseline CA 19-9 values were missing in another 27 patients, and the remaining 121 patients were included in the study (Fig. 1). The study was approved by the responsible ethics committee (permit number 2018-13618).

Contrast-enhanced computed tomography or magnetic resonance imaging prior to and in the course of chemotherapy were evaluated to determine the size and number of intrahepatic lesions, translobar and extrahepatic tumor spread, and presence of nodal and distant metastases. Translobar spread was defined as tumor extension per continuitatem or intrahepatic metastasis in more than one lobe. Extrahepatic spread was defined as a perforation of the liver viscera and/or continuous tumor infiltration in adjacent organs. The International Union against Cancer (UICC) staging system in its current 8th edition was used for tumor staging (Kim et al. 2017). Tumor response was determined according to the current RECIST guidelines (Eisenhauer et al. 2009) through independent examination by two board-certified radiologists (FH and FJ), each with more than five years’ experience in abdominal imaging. In case of discrepancy, a consensus reading was performed.

Serum CA 19-9 levels were collected prior to treatment and after initiation of chemotherapy from the laboratory information system. Data from follow-up visits were extracted from the hospital and radiology information systems. Death dates were queried at the appropriate Resident’s Registration offices.

When investigating the relationship of baseline CA 19-9 values to survival time, the latter was calculated as the time elapsed from the beginning of chemotherapy to death or loss to follow-up. When investigating the predictive power of initial response under chemotherapy, the residual survival time from follow-up CA 19-9 value respectively follow-up imaging to death or loss to follow-up was calculated.

Statistical analyses were performed in R 3.6.1 (R Core Team 2019). Survival analyses were carried out using the packages “survival” and “survminer” (https://cran.r-project.org/package=survival, https://cran.r-project.org/package=survminer, accessed 30.11.2019). Log-rank testing was performed to stratify patients into survival groups. CA 19-9 cut-offs for dichotomization of the cohort were calculated using optimal stratification. Receiver operating characteristics (ROC) analysis was performed using the packages “pROC”, “OptimalCutpoints”, and “plotROC” (https://cran.r-project.org/package=pROC, https://cran.r-project.org/package=OptimalCutpoints, https://cran.r-project.org/package=plotROC, accessed 30.11.2019).

As the intent of this analysis was exploratory, *p*‐values should be interpreted in a descriptive manner. A *p*-value of < 0.05 was considered statistically significant.

## Results

### Baseline characteristics

Median follow up time for all patients was 9.9 months. Administered chemotherapies were gemcitabine-based (*n* = 86, median number of cycles 5), capecitabine-based (*n* = 9, median number of cycles 6), fluorouracil-based (*n* = 8, median number of cycles 12), and others (*n* = 18). Further baseline characteristics are depicted in Table 1.

**Table 1:** Baseline characteristics of patients with intrahepatic cholangiocarcinoma (ICC) prior to systemic chemotherapy

	All (*n* = 121)
Age, years, median (IQR)	63.1 (54–71)
Sex, *n* (%)	
Male	66 (54.5)
Female	55 (45.5)
Sum of intrahepatic lesions, mm, median (IQR)	107 (64–165)
Tumor spread, *n* (%)	
Unifocal or intralobar metastases	58 (47.9)
Translobar metastases	50 (41.3)
Exclusively extrahepatic metastases	13 (10.8)
Distant metastases, *n* (%)	
Negative	43 (35.5)
Positive	78 (64.5)
UICC stage, *n* (%)	
I	11 (9.1)
II	14 (11.6)
III	18 (14.9)
IV	78 (64.4)
CA 19-9 serum levels, U/ml, median (IQR)	49 (19–484)
Previous therapies, *n* (%)	
Resection	47 (38.8)
IAT	9 (7.4)
None	68 (56.2)
Chemotherapy regimen, *n* (%)	
Gemcitabine + cisplatin	48 (39.7)
Other gemcitabine-based	38 (31.4)
Capecitabine-based	9 (7.4)
Fluorouracil-based	8 (6.6)
Others	18 (14.9)
Subsequent therapies, *n* (%)	
Second line chemotherapy	41 (33.9)
IAT	11 (9.1)
SBRT	10 (8.3)

*SD* standard deviation, *IQR* interquartile range, *UICC* International Union against Cancer, *CA *19-9 carbohydrate antigen 19–9, *IAT* intraarterial therapy, *SBRT* stereotactic body radiation therapy

### ***Baseline CA ***19-9 ***values***

**Fig. 2:**
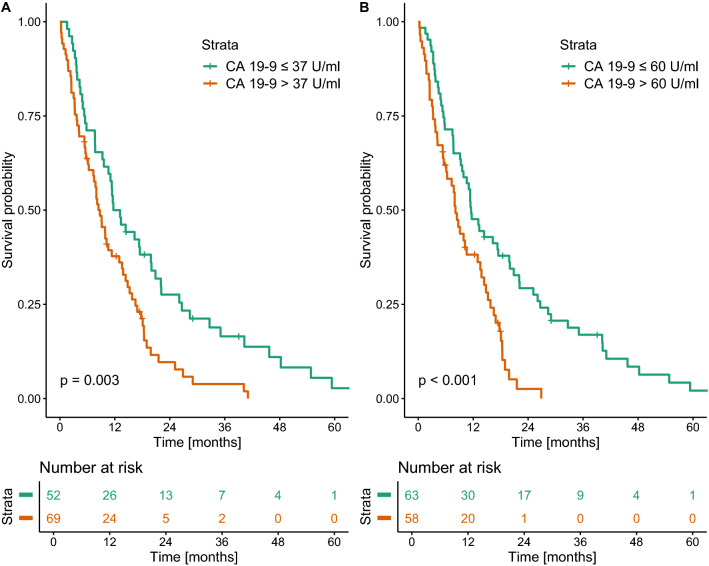
Kaplan–Meier curves of overall survival using baseline CA 19-9 cut-offs at 37 U/ml (**a**) and 60 U/ml (**b**)

Baseline serum CA 19-9 levels above the previously published cut-off of 37 U/ml were associated with poor survival (median OS 8.7 vs. 12.4 months, *p* = 0.003). However, all cut-offs from 22 U/ml to 2092 U/ml were predictive regarding OS and resulted in statistically significant divergence of survival curves. Using optimal stratification, the optimal cut-off was 60 U/ml in our cohort (median OS 8.1 vs. 11.5 months, *p* < 0.001; Fig. 2).

### Initial response to chemotherapy

**Fig. 3:**
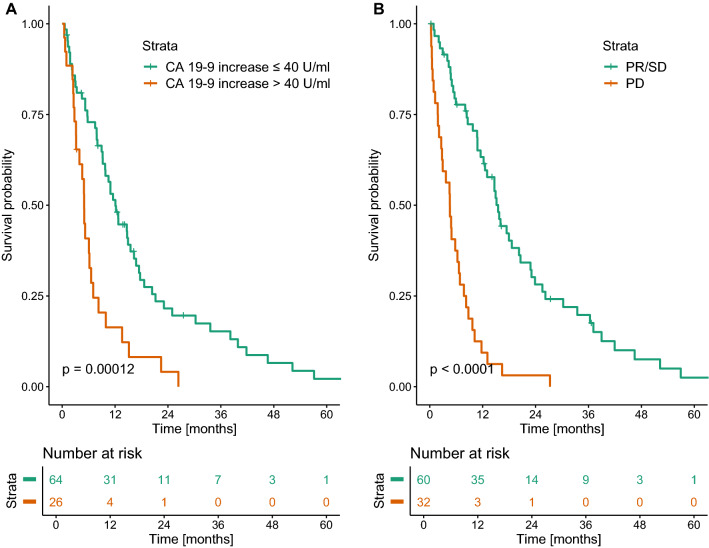
Kaplan–Meier curves of overall survival after chemotherapy start stratified according to absolute CA 19-9 increases (**a**) und response in cross-sectional imaging (**b**) (*PR* partial response, *SD* stable disease, *PD* progressive disease)

Serum CA 19-9 levels within 180 days after the start of chemotherapy were missing in 31 patients; 90 patients had follow-up values within that timeframe, with a median time between start of chemotherapy and first follow-up CA 19-9 value of 60 days (interquartile range 40–91 days). An increase in CA 19–9 > 40 U/ml under chemotherapy was associated with significantly impaired residual survival using optimal stratification (median OS 5.0 vs. 12.1 months, *p* < 0.001; Fig. 3a).

When investigating response in imaging, the median time between the start of chemotherapy and the first follow-up imaging was 66 days (interquartile range 49–90 days). In our cohort, the majority of patients achieved SD under chemotherapy (*n* = 52), followed by patients with PD (*n* = 32). Only a small percentage of patients actually achieved PR under chemotherapy (*n* = 8), and none achieved CR.

PD at the first follow-up investigation despite chemotherapy was associated with significantly worse residual OS compared with patients with SD or PR (median OS 4.6 vs. 15.5 months, *p* < 0.001; Fig. 3b). In case of initial tumor progression (*n* = 32), 11 patients received second-line chemotherapy, 3 patients intraarterial therapies, 3 patients stereotactic body radiation therapy, and 7 patients were switched to best supportive care. Eight patients were initially continued on first-line therapy who had a rather constant tumor load and only radiographic evidence of small new metastases. Among the 29 patients who had no records of performed follow-up imaging, survival was even poorer, with a median survival of only 3.8 months after initiation of chemotherapy.

When using a combination of the above-mentioned factors and classifying patients with either progressive disease in imaging or an increase in CA 19-9 of more than 40 U/ml as tumor progression, stratification of patients into risk groups only marginally improved compared to the monofactorial approach (median OS 4.6 months vs 15.7 months, *p* < 0.001, Fig S1).

### Correlation between CA 19-9 changes and imaging response

After initiation of chemotherapy, follow-up serum CA 19-9 levels and imaging within 28 days of each another were available for 66 patients.

**Fig. 4:**
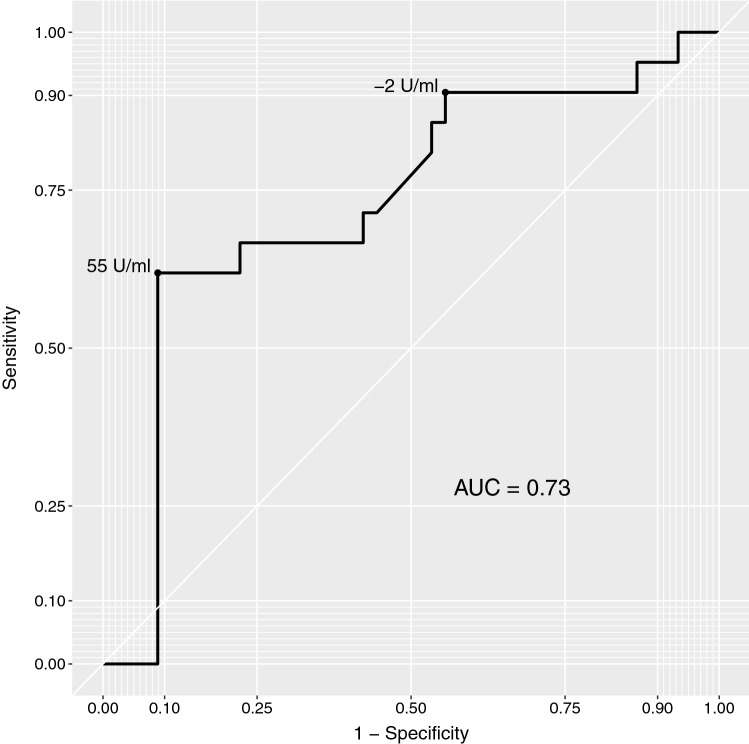
ROC analysis comparing changes in serum CA 19-9 levels with progressive disease in follow-up imaging (AUC, area under the curve)

The results of ROC analysis investigating changes in serum CA 19-9 values with respect to progressive disease in follow-up imaging are depicted in Fig. 4 and yielded an area under the curve of 0.73. The contingency table that resulted from using optimal cut-offs with regard to the positive and negative predictive values of changes in serum CA 19-9 level and imaging response is depicted in Table 2.

**Table 2:** Contingency table showing changes in serum CA 19-9 levels and response in cross-sectional imaging

	PR	SD	PD	Sum
CA 19-9 decrease > 2 U/ml	3	17	2	22
CA 19-9 change between − 2 to 55 U/ml	3	18	6	27
CA 19-9 increase > 55 U/ml	0	4	13	17
Sum	6	39	21	66

*PR* partial response, *SD* stable disease, *PD* progressive disease

Here, an increase of CA 19-9 above 55 U/ml resulted in a positive predictive value for progressive disease in imaging of 76%, whereas a decrease in CA 19-9 of more than 2 U/ml resulted in a negative predictive value of 91%. However, 27 of 66 patients (41%) showed intermediate changes in serum CA 19-9 levels, from − 2 to 55 U/ml, with no clear association between CA 19-9 changes in this range and response in imaging.

## Discussion

In our study, baseline serum CA 19-9 levels prior to chemotherapy were predictive of poor survival. During the course of chemotherapy, an initial increase in CA 19-9 outperformed baseline serum levels with regards to survival stratification. PD in follow-up imaging showed the best predictive performance towards residual OS in our cohort.

While several studies have previously shown prognostic value in baseline CA 19-9 serum levels with regard to survival (Ali et al. 2007; Bolm et al. 2019; Coelho et al. 2017; He et al. 2018; Jiang et al. 2011), studies investigating CA 19-9 kinetics in patients with ICC undergoing chemotherapy are scarce. A study by Harder et al. investigated 70 patients with biliary tract cancers, among them 33 patients with ICC. In their cohort, patients with a decrease in CA 19-9 levels after treatment showed an improved survival, without specifying a cut-off value (Harder et al. 2007). Lee et al. investigated a cohort of 179 patients, including 97 with ICC. They found a decline of at least 50% in CA 19-9 level to be the most significant prognostic factor within their entire cohort, with a median survival time of 16.0 months vs. 9.0 months (Lee et al. 2016). Our data did not show statistically relevant differences in survival times with respect to absolute or relative decreases in serum CA 19-9 levels.

Previous studies have suggested that tumor location influences the effectiveness of chemotherapy, with patients affected by distal cholangiocarcinoma showing a larger benefit (Serafini et al. 2001), while the survival benefit of chemotherapy in the management of patients with ICC is limited (Rahnemai-Azar et al. 2017). In the study by Lee et al., perihilar and distal cholangiocarcinomas also showed lower hazard ratios in hazard regression analysis than ICC (Lee et al. 2016). This might explain the findings in our study, which focused exclusively on patients with ICC. In the studies by Harder and Lee, ICCs constituted only 47% and 54% of the tumors, respectively (Harder et al. 2007; Lee et al. 2016). Therefore, the better overall response to chemotherapy they observed might be due to the number of perihilar and distal cholangiocarcinomas.

In contrast to Harder et al. and Lee et al., we found that the disease control rate, comprising CR, PR, and SD—that is, the absence of progressive disease—was the strongest predictor of prolonged residual OS in our cohort. PD as the best predictor of poor survival in initial follow-up imaging has been observed in other aggressive tumor entities: Lara et al. investigated survival in patients with advanced non-small cell lung cancer under platinum-based chemotherapy, and found that differentiating between PD and the disease control rate at week 8 was the strongest predictor of subsequent survival outcome (Lara et al. 2008).

Moreover, the positive predictive value of increasing serum CA 19-9 levels and negative predictive values of decreasing serum CA 19-9 levels with regard to PD and the disease control rate in imaging response were 76% and 91%, respectively. Therefore, laboratory markers have the potential to detect at-risk patients who are likely to benefit from closer imaging surveillance.

In our cohort, patients with CA 19-9 changes from − 2 to 55 U/ml constituted a subgroup with heterogeneous imaging responses and progressive disease in 22% of patients. However, differentiating viable tumor tissue and tumor necrosis under chemotherapy can be challenging, especially in liver tumors, and thus imaging response may be misleading. This has led to the development of modified RECIST (mRECIST) for hepatocellular carcinoma and the Choi criteria for gastrointestinal stromal tumors (Choi et al. 2004; Lencioni et al. 2017). In the absence of a specific response evaluation system for ICC, we decided to use RECIST in its current version because of its widespread use (Eisenhauer et al. 2009). Despite these shortcomings, PD in follow-up imaging was the best predictor of residual OS in our study.

As intrahepatic cholangiocarcinoma is a rare tumor entity, the vast majority of studies investigating systemic chemotherapies are retrospective (Chun and Javle 2017). This holds true for our analysis as well, and the times at which CA 19-9 assays and imaging follow-ups were performed were not standardized. However, the median times between the start of chemotherapy and the first follow-up investigation were 60 and 66 days for CA 19-9 and imaging, respectively, corresponding to the end of the third cycle in the gemcitabine-based chemotherapy regimen (cycle length 21 days) (Valle et al. 2010).

CA 19-9 laboratory testing is less costly and more convenient than cross-sectional imaging; however, the information obtained by imaging evaluation exceeds the information obtained by the tumor marker considerably. Knowledge of the site of tumor progression has direct impact on further treatment options as it is essential to devise optimal treatment (Rizvi et al. 2018). Moreover, false-positive CA 19-9 values when diagnosing patients with cholangiocarcinoma have been reported in over 15% of cases (Qin et al. 2004) and false positive CA 19-9 values due to concomitant inflammation or stenosis may lead to an increased distress in patients during follow-up.

Our analysis has several further limitations. First, the study was single-centered and conducted retrospectively. Second, the sample size (*n* = 121) was moderate. However, due to the low incidence of ICC in western countries, prior studies on the kinetics of ICC had to cope with similar or even considerably smaller sample sizes. Moreover, as gemcitabine in combination with cisplatin became the current standard in 2010, the cohort was heterogeneous in terms of chemotherapy regimens due to the long recruitment period. Multidisciplinary, multi-center efforts are needed in further studies to achieve larger cohorts and allow for prospective studies.

## Conclusion

In our study, the disease control rate (the absence of progressive disease) was the strongest predictor of prolonged residual OS in our cohort. Changes in CA 19-9 values and progressive disease on initial follow-up both showed remarkable discriminatory power, with PD slightly outperforming CA 19-9. Therefore, imaging should remain the mainstay of patient evaluation during follow-up. However, changes in serum CA 19-9 levels have the potential to identify patients who are at risk for progressive disease and likely to benefit from closer imaging evaluation.

## Electronic supplementary material

Below is the link to the electronic supplementary material.

Supplementary file1 (TIFF 875 kb) Fig. S1 Kaplan-Meier curves of overall survival after chemotherapy start stratified according to response in cross-sectional imaging (a) and using either imaging or tumor marker progress as indicator for tumor progression (b) (PR, partial response; SD, stable disease; PD, progressive disease)
